# Reactive Oxygen Species and Strategies for Antioxidant Intervention in Acute Respiratory Distress Syndrome

**DOI:** 10.3390/antiox12112016

**Published:** 2023-11-18

**Authors:** Eun Yeong Lim, So-Young Lee, Hee Soon Shin, Gun-Dong Kim

**Affiliations:** 1 Division of Food Functionality Research, Korea Food Research Institute (KFRI), Wanju 55365, Republic of Korea; l.eunyeong@kfri.re.kr (E.Y.L.); sylee09@kfri.re.kr (S.-Y.L.); hsshin@kfri.re.kr (H.S.S.); 2 Department of Food Biotechnology, Korea University of Science and Technology (UST), Daejeon 34113, Republic of Korea

**Keywords:** acute respiratory distress syndrome, reactive oxygen species, antioxidant, acute lung injury, superoxide dismutase, glutathione, vitamins

## Abstract

Acute respiratory distress syndrome (ARDS) is a life-threatening pulmonary condition characterized by the sudden onset of respiratory failure, pulmonary edema, dysfunction of endothelial and epithelial barriers, and the activation of inflammatory cascades. Despite the increasing number of deaths attributed to ARDS, a comprehensive therapeutic approach for managing patients with ARDS remains elusive. To elucidate the pathological mechanisms underlying ARDS, numerous studies have employed various preclinical models, often utilizing lipopolysaccharide as the ARDS inducer. Accumulating evidence emphasizes the pivotal role of reactive oxygen species (ROS) in the pathophysiology of ARDS. Both preclinical and clinical investigations have asserted the potential of antioxidants in ameliorating ARDS. This review focuses on various sources of ROS, including NADPH oxidase, uncoupled endothelial nitric oxide synthase, cytochrome P450, and xanthine oxidase, and provides a comprehensive overview of their roles in ARDS. Additionally, we discuss the potential of using antioxidants as a strategy for treating ARDS.

## 1. Introduction

Acute respiratory distress syndrome (ARDS) imposes a huge burden in the context of critical care medicine, demanding immediate attention and a comprehensive approach due to its life-threatening nature. Initially described in the 1960s by Petty and Ashbaugh [1], ARDS is characterized by the sudden onset of tachypnea, hypoxemia, and reduced lung compliance. The absence of a standardized or precise definition for ARDS has posed limitations in patient treatment and the statistical analysis of clinical outcomes [2]. In response to this challenge, the Berlin definition was introduced in 2012 as a novel diagnostic framework for ARDS [3]. This definition includes criteria such as (I) acute onset within 1 week; (II) bilateral lung infiltrates observable upon chest radiography; (III) the absence of a fully explanatory cause related to cardiac failure or fluid overload; and (IV) the categorization of ARDS severity based on a positive end-expiratory pressure (PEEP) level of at least 5 cm H_2_O, with lung injury classified into three grades of severity according to the PaO_2_/FiO_2_ ratio: mild (201–300 mm Hg), moderate (101–200 mm Hg), and severe (≤100 mm Hg). Historically, acute lung injury (ALI) referred to a milder form of lung injury, while ARDS signified a more severe lung condition [4]. However, the clinical use of the term ALI has been discontinued, and the classification is now based on the severity of ARDS, providing a clear and consistent categorization for clinical purposes.

ARDS can be attributed to various conditions, with trauma, aspiration, pneumonia, and sepsis serving as primary triggers for its development [5,6,7]. Additionally, the inhalation of harmful substances, aspiration of vomit, pancreatitis, blood transfusion, and near-drowning episodes can also induce ARDS. Recent retrospective observational studies have revealed that 67% of patients with severe COVID-19 develop ARDS, further emphasizing its significance as a primary cause of mortality [8]. Presently, ARDS stands as a leading cause of morbidity and mortality, with no effective treatments available [9]. Therefore, there is an urgent need to elucidate the pathogenesis of ARDS and develop innovative treatment strategies.

### 1.1. ARDS Incidence and Mortality

The incidence of ARDS varies globally, with data from population-based studies revealing significant diversity [9,10]. A study conducted in the United States from 1999 to 2000 reported an incidence rate of 78.9 per 100,000 person-years [11], while research in Iceland from 1988 to 2010 indicated incidence rates ranging from 3.65 to 9.63 [12]. These findings provide insights into mortality rates and severity distribution among patients with ARDS. In a large international, multicenter, prospective cohort study performed over a period of 4 weeks in 2014, patients with ARDS accounted for 10.4% of the total sample, with the proportions of mild, moderate, and severe cases being 30%, 46.5%, and 23.4%, respectively [13]. The mortality rates, according to severity, were 34.9% for mild, 40.3% for moderate, and 46.1% for severe. Another study conducted in China from March 2016 to February 2018 across 18 intensive care units (ICUs) reported that 3.6% of a total of 18,793 patients were diagnosed with ARDS [14]. Among these patients, 9.7% had mild ARDS, 47.4% exhibited moderate ARDS, and a substantial 42.9% were classified as having severe ARDS, with a reported mortality rate of 46.3%. Systematic research conducted since 2010 has indicated overall in-hospital, ICU, 28/30-day, and 60-day mortality rates of 45%, 38%, 30%, and 32%, respectively [15]. Multiple studies have highlighted the correlation between ARDS severity, high mortality, and prolonged ventilation duration.

### 1.2. ARDS Pathophysiology

Under normal physiological conditions, lung fluid balance is maintained through fluid clearance. However, dysfunction of the alveolar barrier can impair alveolar fluid clearance, a prominent hallmark of patients with ARDS, leading to hypoxemia and pulmonary bilateral edema [16,17]. Additionally, ARDS is characterized by an increased production of pro-inflammatory factors and an elevated expression of adhesion molecules, which play a crucial role in the recruitment of leukocytes. Specifically, activated neutrophils contribute to tissue damage by releasing cytotoxic agents, including granular enzymes, pro-inflammatory cytokines, bioactive lipids, and reactive oxygen species (ROS). Typical ARDS symptoms include dyspnea, an increased respiratory rate, tachycardia, and cyanosis. The pattern of injury observed in patients with ARDS is not uniform, and clinical symptoms can vary according to the severity or stage [18]. ARDS can be categorized into discrete stages, each with temporal evolution, pathophysiological alterations, histological features, and clinical implications. These stages encompass three simultaneous phases: exudative, proliferative, and fibrotic [19,20].

The acute phase, referred to as the exudative stage, typically spans from hours to the first week after the injury onset. During this stage, a cascading inflammatory response is activated, leading to an influx of neutrophils [21], a phenomenon that results in the disruption of the alveolar epithelial and endothelial barriers, and consequently, protein-rich edema in the interstitium and alveolar spaces. Hyaline membranes, composed of dead cells, surfactants, and plasma proteins, are also characteristic of diffuse alveolar damage [22]. The subsequent proliferative stage involves an organization and repair process that includes the proliferation of fibroblasts, primarily within the interstitium. In the late proliferative stage, most hyaline membranes are resorbed, although some remnants may persist along the alveolar septa [23]. Early fibrotic changes and thickening of the alveolar capillaries can also be observed. Finally, the fibrotic stage is marked by an increased deposition of collagen and enlarged air spaces, although not all patients progress to this stage. Established fibrosis reduces lung compliance, resulting in increased breathing effort, which is associated with poor lung function and a high risk of mortality [19].

To gain a deeper understanding of the intricate pathophysiology of ARDS and to develop novel therapeutic strategies, researchers have extensively utilized controlled experimental models to mimic clinical syndromes. These models serve as invaluable tools for exploring the mechanisms underlying ARDS and evaluating potential interventions. The workshop report published in 2011 by the American Thoracic Society (ATS) proposed the following four features of ARDS for preclinical models: histological evidence of tissue injury, alterations in the alveolar-capillary barrier, inflammatory response, and physiological dysfunction [24]. Histological evidence of tissue injury includes the infiltration of neutrophils in the alveolus or interstitium, the presence of hyaline membranes, and the accumulation of proteinaceous debris in the alveolus, along with thickening of the alveolar wall. Alveolar-capillary barrier disruption is reflected in increased extravascular lung water content, bronchoalveolar lavage fluid (BALF) protein levels, and microvascular filtration coefficient, and the accumulation of an exogenous tracer. The inflammatory response is characterized by an increase in the number of inflammatory cells in the BALF, lung myeloperoxidase (MPO) activity, BALF protein concentration, and pro-inflammatory cytokines in the lung or BALF. Lastly, physiological dysfunction comprises hypoxemia and an increased alveolar-arterial oxygen difference. To ensure ARDS in preclinical models, at least three of these four criteria should be fulfilled.

ARDS models have been generated through both direct and indirect methods to recapitulate human ARDS. The direct approach involves the stimulation of conditions such as pneumonia and acid aspiration, while the indirect approach involves the stimulation of pancreatitis and non-pulmonary sepsis. In direct models, ARDS is induced through the intranasal or intratracheal administration of agents such as lipopolysaccharides (LPS), HCl, bleomycin, bacteria, and viruses. Hyperoxia is also a direct cause of ARDS. In contrast, indirect lung injury can be induced via the intravenous injection of LPS, cecal ligation and puncture (CLP) to induce sepsis, hemorrhagic shock, mesenteric ischemia, and reperfusion.

#### 1.2.1. LPS-Induced Sepsis Model

The most frequently utilized ARDS model involves the instillation of LPS via either the intratracheal or intranasal routes. Research has also explored variations based on species, age, sex, and LPS concentrations. In the LPS-induced ARDS model, several key observations include a reduced PaO_2_/FiO_2_ ratio, increased total protein concentration in the BALF, infiltration of inflammatory cells, disruption of alveolar barrier function, and excessive generation of ROS and oxidative stress [25,26,27]. These changes are often accompanied by local hemorrhage and edema [28]. However, the limitations of this model include the fact that alveolar and interstitial edema is mild, hyaline membrane formation is absent, and the decrease in PaO_2_ is not sustained for an extended period [29]. These limitations can be mitigated by employing a two-hit approach involving LPS stimulation, where both intratracheal LPS and intravenous LPS are administered [29]. This two-hit model involves the intraperitoneal injection of a small dose of LPS (1 mg/kg), followed by tracheal instillation with a moderate dose of LPS (5 mg/kg). In this model, the decrease in PaO_2_ is sustained for more than 72 h and induces more pronounced hyaline membrane formation and edema. Additionally, several studies have reported that the intraperitoneal injection of LPS can lead to lung injury [30,31].

#### 1.2.2. Acid Aspiration Model

In the clinical setting, the aspiration of gastric contents is a major contributor to ARDS. Given the role of pH in lung injury, hydrochloric acid (HCl) has been employed to mimic human ARDS. The orotracheal and intratracheal instillation of HCl leads to reduced oxygenation, increased respiratory elastance, and pulmonary inflammation [32]. Furthermore, HCl aspiration induces histological changes, lung edema, and inflammatory responses in rabbits [33].

#### 1.2.3. Oleic Acid Injection Model

The intravenous injection of oleic acid induces ARDS characterized by intra-alveolar edema and an increased infiltration of inflammatory cells [34,35]. An advantage of the oleic acid method is the rapid and reversible development of sparse inflammatory lung injury [36]. In research involving pigs, the administration of oleic acid resulted in a high mortality rate and extensive histological changes, as well as elevated levels of IL6 and IL8 [37]. Additionally, the intratracheal injection of oleic acid, employed in a mouse ARDS model, resulted in elevated neutrophil and protein levels in the BALF, the activation of leukocytes, and the production of inflammatory mediators [38].

#### 1.2.4. CLP-Induced Sepsis Model

CLP is a method commonly utilized to induce sepsis in models [39]. In brief, in this method, the cecum is ligated, punctured with a needle, and compressed to expel a small amount of feces [39]. The severity of the injury can vary depending on factors such as the percentage of ceca ligated, the number of punctures, and the size of the needle used. Puncturing the cecum, which contains bacteria, leads to bacterial peritonitis and the translocation of bacteria into the bloodstream, ultimately resulting in septic shock. CLP mice exhibit high mortality and histopathological changes associated with ARDS, including the destruction of the alveolar structure, thickening of the pulmonary septa, hemorrhaged lung tissue, and the infiltration of inflammatory cells [40,41].

### 1.3. Treatment

The primary goal of managing ARDS is to improve blood oxygen levels. Despite administering high levels of inspired oxygen, oxygen saturation often remains inadequately low, underscoring the severity of impaired gas exchange or the failure to respond to conventional respiratory therapy in patients with ARDS. Furthermore, due to the diversity of ARDS causes and the complexity of the syndrome, there is currently no single therapy tailored specifically to ARDS. Additionally, a decline in the quality of life is frequently observed even after recovering from ARDS.

Various professional societies, including the Faculty of Intensive Care Medicine and Intensive Care Society [42], The French Society of Intensive Card Medicine (Société de Réanimation de Langue Française) [43], The ATS, the European Society of Intensive Care Medicine [44], the Korean Society of Critical Care Medicine and Korean Academy of Tuberculosis and Lung Diseases of South Korea [45], the Society of Critical Care Medicine, and the World Health Organization, offer recommendations for managing ARDS, taking into account the patient’s condition. These guidelines have been thoroughly documented in review papers [46,47] and propose various management strategies for patients with ARDS; these strategies can be categorized into two main groups: (1) ventilation strategies, including non-invasive ventilation, low tidal volume ventilation (LTVV), high positive end-expiratory pressure (PEEP) strategies, recruitment maneuvers, and high-frequency oscillatory ventilation, and (2) non-ventilation strategies, including interventions such as neuromuscular blockade, inhaled vasodilators, corticosteroids, and other pharmacological agents [47,48].

Mechanical lung ventilation is a standard therapeutic approach used to enhance oxygenation in patients with ARDS. However, it can also lead to ventilation-induced lung injury (VILI) [49,50]. VILI manifests as clinical symptoms such as shallow breathing, respiratory distress, and cyanosis, as well as chest X-ray findings indicating bilateral lung infiltrates, reduced blood oxygen levels (hypoxemia), and impaired lung elasticity, among other pulmonary functional impairments. Minimizing VILI through mechanical ventilation and effectively managing refractory hypoxemia are critical aspects of supportive ARDS management.

VILI is characterized by four primary mechanisms: volutrauma, barotrauma, atelectrauma, and biotrauma [49]. Volutrauma is closely linked to the use of high tidal volumes during mechanical ventilation. When mechanical ventilation is applied with a typical tidal volume without considering collapsed lung regions, it can result in the overdistension of lung cells, ultimately leading to increased alveolar-capillary permeability and alveolar destruction, culminating in pulmonary edema. Barotrauma arises from high airway pressure during positive pressure ventilation, causing lung damage through overinflation and the rupture of lung cells. The repeated opening and closing of lung cells contributes to increased lung size and shear stress forces, resulting in lung injury and surfactant dysfunction, known as atelectrauma. Biotrauma refers to the biochemical processes that occur when mechanical forces cause the collapse of lung cell membranes, triggering an inflammatory response. This can lead to systemic inflammatory response syndrome and multiple organ failure, ultimately resulting in death. Importantly, these four mechanisms are interconnected and can mutually influence each other. Various lung-protective ventilation methods are employed to minimize VILI.

#### 1.3.1. Low Tidal Volume Ventilation

Patients with ARDS often exhibit reduced lung volume due to edema and inflammation, colloquially referred to as the “baby lung”. Consequently, traditional tidal volume ventilation (10 mL/kg or more) can lead to lung overdistension in patients with ARDS. LTVV is a technique that involves reducing the tidal volume, a strategy commonly used in individuals with healthy lungs to prevent lung tissue overdistension [17]. In 1998, Amato et al. first reported the potential clinical benefits of LTVV in patients with ARDS [51]. An extensive ARDS Network study involving 861 patients demonstrated that LTVV (6 mL/kg of predicted body weight) reduced mortality from 39.8% to 31% compared to traditional tidal volume ventilation (12 mL/kg of predicted body weight) and also increased the number of ventilator-free days [52]. Recent research suggests that the effects of mechanical ventilation can be enhanced with different tidal volumes (depending on lung compliance), with patients exhibiting low respiratory system compliance being more vulnerable to damage at higher driving pressures [53]. Furthermore, patients with COVID-19 receiving LTVV have shown reduced 28-day mortality [54].

#### 1.3.2. Positive End-Expiratory Pressure

PEEP involves maintaining a pressure higher than atmospheric pressure at the end of expiration, which helps prevent atelectrauma and corrects hypoxemia resulting from alveolar hypoventilation [55]. Physiologically, PEEP increases functional residual capacity, recruits collapsed alveoli, and redistributes fluid to the interstitial space to improve oxygenation [56]. These physiological effects are expected to reduce VILI. However, it has been reported to have side effects, including a decrease in cardiac output and the potential for barotrauma. Studies on the effectiveness of high levels of PEEP in patients with ARDS have yielded inconsistent results. Some meta-analyses suggest that higher PEEP can enhance oxygenation during the first and third days of ventilation [57]. However, other meta-analyses have indicated that there is no significant difference in the 28-day mortality between high- and low-PEEP groups [58,59]. Additional studies are needed to accurately determine the impact and outcomes of PEEP application in patients with ARDS.

#### 1.3.3. Lung Recruitment Maneuvers (LRMs)

LRMs are strategies designed to transiently increase the driving pressure to facilitate the recruitment of collapsed alveoli. This recruitment process improves oxygenation, promotes uniform volume distribution in the lungs, and mitigates the risk of overdistension [60]. Sustained inflation is employed at pressures ranging from 35 to 50 cm H_2_O for 20 to 40 s, with continuous monitoring for any adverse effects [61,62]. The use of LRMs in the treatment of ARDS remains a subject of debate. A meta-analysis that included data from 10 trials involving 1658 patients indicated that the implementation of LRMs in patients with ARDS led to reduced ICU mortality [63]. However, this study had limitations, primarily due to co-interventions with other mechanical ventilation strategies. Conversely, a more recent study with 10 trials involving 3025 patients suggested that LRMs did not affect the mortality of patients with ARDS, only reducing the duration of hospital stay [64]. Further research is necessary to clarify the precise impact and potential benefits of the isolated application of LRMs on the overall management and outcomes of patients with ARDS.

#### 1.3.4. Prone Position

The prone position is commonly employed to facilitate breathing in ventilated patients. It involves turning the patient from the supine (back) position to the prone (face down) position to improve oxygenation. ARDS patients in the supine position are affected by the weight of the heart and abdominal organs, leading to increased pleural pressure in the dorsal lung regions, resulting in lung collapse. In such cases, the leveraging of the prone position can help redistribute blood and airflow more evenly [65,66]. The Prone Positioning in Severe ARDS (PROSEVA) study in 2013 demonstrated that employing the prone position during ventilation reduced both the 28- and 90-day mortality rates [67]. A meta-analysis of eight randomized controlled trials (RCTs) involving 2129 patients conducted by Munshi et al. suggested that employing the prone position for more than 12 h daily is likely to reduce mortality among patients with severe ARDS [68]. Furthermore, the PaO_2_/FiO_2_ ratio was significantly higher in the prone position group on day 4. A recent meta-analysis also indicated that prone positioning during venovenous extracorporeal membrane oxygenation improved survival [69,70]. Nevertheless, PEEP in the prone position is approached with caution due to potential adverse effects, including endotracheal tube obstruction, pressure sores, and facial edema [71].

#### 1.3.5. Corticosteroids

Corticosteroids are potent anti-inflammatory and immunomodulatory drugs commonly employed to treat various inflammatory diseases, including asthma, allergic rhinitis, chronic obstructive pulmonary disease (COPD), and pneumonia [72]. The use of corticosteroids in patients with ARDS has yielded varying results. A meta-analysis of corticosteroid use in ARDS encompassing seven RCTs involving 851 patients indicated that corticosteroids reduced mortality and the duration of mechanical ventilation while increasing the number of ventilator-free days [73]. Furthermore, another meta-analysis demonstrated that patients with ARDS, both with COVID-19 and non-COVID-19 cases, exhibited reduced mortality and decreased mechanical ventilation duration when treated with corticosteroids [74]. However, a separate meta-analysis found that corticosteroids reduced mortality in non-COVID-19 patients with ARDS but had no effect in the COVID-19 subgroup [75]. It is important to exercise caution when using corticosteroids, as they may be associated with a higher mortality rate or the development of hyperglycemia [73,76].

## 2. Oxidative Stress and Lung Injury

### 2.1. Generation of ROS in ARDS

**Figure 1:**
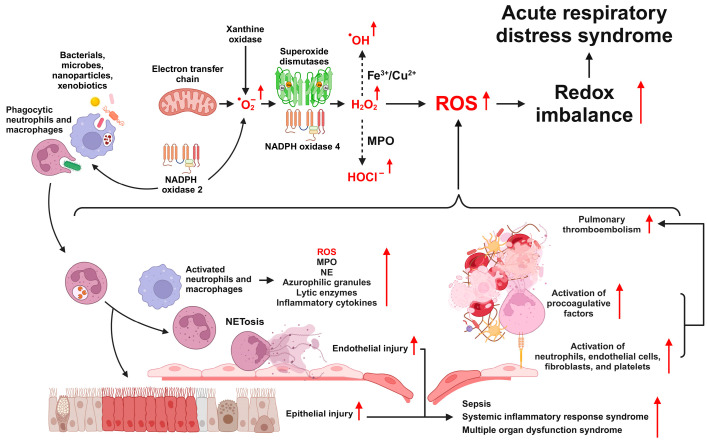
Overview of the roles of reactive oxygen species (ROS) in the pathophysiology of ARDS. ROS originating from various intrinsic and extrinsic sources leads to oxidative stress, cell-mediated inflammation, and damage to epithelial and endothelial cells. These processes are driven by the activation of multiple signaling pathways, ultimately contributing to the onset and progression of ARDS.

The overproduction and accumulation of ROS in ARDS can be attributed to several key factors, including the accumulation of leukocytes, elevated levels of nicotinamide adenine dinucleotide phosphate (NADPH) oxidases (NOXs) and xanthine oxidase (XO), and the use of high-concentration oxygen during mechanical ventilation. A defining characteristic of ARDS is the excessive infiltration of neutrophils and macrophages into the lung tissue [47,77]. Leukocytes play an essential role in the host defense against pathogens and contribute to the initiation and amplification of inflammatory responses by migrating to sites of infection and inflammation, engaging in phagocytic activity, and producing inflammatory mediators [78]. Neutrophils, monocytes, and macrophages employ NOX2 to generate MPO and ROS during their phagocytic actions (Figure 1). Furthermore, the presence of ROS is closely linked to the prognosis of sepsis, a primary cause of ARDS [79]. Contemporary research focuses on understanding the mechanisms underlying ARDS and exploring treatments using various preclinical models. Numerous studies have documented ROS generation in ARDS models and have shown that reduced ROS production can alleviate the lung damage associated with ARDS (refer to Table 1 for details).

LPS treatment increases the uncoupling of eNOS and mitochondrial ROS production, resulting in the activation of NLR family pyrin domain containing 3 protein (NLRP3) inflammasome in human lung microvascular endothelial cells [9]. The inhibition of eNOS uncoupling can reduce ROS production and NLRP3 inflammasome levels. Targeting mitochondrial ROS using antioxidants can alleviate lung injury in the ARDS model. The mitochondrial redistribution of uncoupled eNOS has been implicated in the mitochondrial ROS-dependent activation of the NLRP3 inflammasome during sepsis.

Xanthine oxidoreductase (XOR) directly transfers electrons to O_2_, generating ROS, including O_2_·^−^ and H_2_O_2_ [90]. Notably, O_2_·^−^ reacts with NO, contributing to the formation of reactive nitrogen species such as peroxynitrite [91]. Grum et al. [92] showed that there was a significant increase in plasma XO activity (approximately 90-fold higher) in the group of patients with ARDS, accompanied by sepsis or pneumonia, compared to patients without ARDS. Additionally, plasma levels of hypoxanthine were approximately twice as high in the ARDS group compared to those in the non-ARDS group. A recent study reported that the myeloid-specific deficiency of XOR suppressed LPS-induced macrophage-mediated inflammation and ROS production while improving mitochondrial respiration in an ARDS model [93]. Febuxostat, a potent XO inhibitor, demonstrated dose-dependent protection against LPS-induced lung inflammation in rats, resulting in decreased levels of tumor necrosis factor alpha (TNFα) and malondialdehyde (MDA), as well as increased superoxide dismutase (SOD) activity in the lung tissue of rats [94]. The intratracheal administration of LPS significantly reduced the inflammatory response in myeloid-specific XOR knockout mice [93]. In addition, treatment with the ROS scavenger N-acetylcysteine (NAC) and XOR knockout significantly reduced NLRP3 and IL1β expression in purified BAL macrophage culture. The inhibition of NOXs also demonstrates positive effects against LPS-induced lung injury. Wang et al. [95] showed that the pharmacological inhibition of NOXs suppressed LPS-induced neutrophil ROS production and septic lung injuries in guinea pigs. Additionally, a recent study reported that increased lipid oxidation, MPO activity, and inflammatory responses were attenuated and antioxidant enzyme activity was restored through the inhibition of NOXs in a murine lung inflammation model [96].

Supplementation with oxygen is a potential treatment option for patients with ARDS [97]. However, prolonged exposure to high oxygen levels (O_2_ > 65%) can lead to an increase in ROS, inducing oxidative stress, worsening respiratory symptoms, and potentially causing lung injury [27]. Consistently, Kim et al. [26] showed that ROS generation significantly increased in type II alveolar epithelial cells and a model of the excessive hyperoxia-induced murine lung inflammation. Pretreatment with cimetidine, a cytochrome P450 (CYP) inhibitor, has been shown to attenuate hyperoxia-induced lung injury in lambs [98]. However, hyperoxic conditions induce cell death and ROS production in BEAS-2B cells [99]. In addition, hyperoxic conditions downregulated CYP1B1 in BEAS-2B cells, suggesting that CYP1B1 does not contribute to an increase in ROS. The siRNA-induced downregulation of CYP1B1 rescued cytotoxicity, whereas the overexpression of CYP1B1 promoted hyperoxic cytotoxicity.

### 2.2. Types and Functions of ROS

ROS are generated when O_2_ gains electrons. Under normal conditions, a stable equilibrium exists between ROS production and elimination. ROS include H_2_O_2_, ^•^OH, 1O_2_, and O_2_^•−^. O_2_^•−^ is generated through a single electron transfer to O_2_ and subsequently dismutates into H_2_O_2_, which is either spontaneously generated in the presence of water or catalyzed by SOD [100]. Notably, although ROS are viewed as a unified entity, different ROS types have distinct targets and activities. In cells, O_2_^−^ and H_2_O_2_ are the two most abundant ROS subspecies, differing significantly in chemical properties and, consequently, behavior and function. O_2_^•−^ is highly reactive and contributes to oxidative stress and cellular damage characterized by protein and amino acid oxidation, DNA damage, and lipid peroxidation. In contrast, H_2_O_2_, a relatively stable molecule, serves as a crucial cellular signaling factor. However, excessive production can lead to cytotoxicity.

### 2.3. Sources of ROS Production

ROS are primarily generated during tightly regulated enzymatic processes involving endothelial nitric oxide (NO) synthase, the mitochondrial respiratory chain, various NOX isoforms, XOs, and CYP enzymes [90,101]. Among these, NOXs appear to be the predominant source of ROS in the vasculature.

#### 2.3.1. NAPDH Oxidase

NOXs are enzymes that oxidize NADH or NADPH to form NAD^+^ or NADP^+^. They catalyze the reduction of oxygen to produce O_2_^•−^ and are known to activate other ROS sources, such as endothelial NO synthase (eNOS). Initially, NOX was discovered in phagocytic immune cells; however, it was later found in non-phagocytic cells. Activated NOX generates ROS, leading to endothelial cell dysfunction. The NOX family comprises seven isoforms: NOX1, NOX2, NOX3, NOX4, NOX5, dual oxidase 1 (Duox1), and Duox2. NOX2 and NOX4 play crucial roles in ROS production. In a spinal cord injury model, ROS had a harmful impact, contributing to oxidative stress, which is reversed by the deletion of NOX2 or inhibition of NOX4 to reduce ROS production and oxidative stress markers [102].

NOX2, in particular, comprises four cytoplasmic components, including p47^phox^, p67^phox^, p40^phox^, and Rac family small GTPase 2, along with transmembrane proteins p22^phox^ and gp91^phox^ [103,104]. Upon the initiation of phagocytosis, the cytosolic subunit Rac2, bound to guanosine diphosphate, becomes activated by a Rac guanine nucleotide exchange factor, allowing it to bind to GTP [105]. Simultaneously, it facilitates the transition of the transmembrane heterodimer p22^phox^ and gp91^phox^ to the phagosomal membrane and induces association with the trimeric complex composed of p40^phox^, p47^phox^, and p67^phox^ [106]. Through the phosphorylation of p47^phox^ and p67^phox^, a structural alteration and stabilization mediated by GTP-Rac2, p22^phox^, and gp91^phox^ take place, inducing the overproduction of O_2_^•−^ and MPO, which exists in the form of a ferric (Fe III) heme enzyme in lysosomes of phagocytic cells and chlorinates H_2_O_2_ to highly reactive hypochlorous acid (HOCl^−^) [103,106]. This, in turn, contributes to the sequential generation of ROS such as H_2_O_2_, ^•^OH, and HOCl^−^ [107].

#### 2.3.2. Uncoupled Endothelial NO Synthase

Under normal circumstances, eNOS generates NO. eNOS transfers the electrons donated by NADPH to the amino-terminal oxygenase domain of heme via a series of cofactors located in the carboxy-terminal reductase domain. These cofactors include flavin adenine dinucleotide and flavin mononucleotide, which facilitate the electron transfer process necessary for the enzymatic conversion of l-arginine into NO. However, under conditions of oxidative stress, it shifts its production toward O_2_^•−^ instead of NO; this phenomenon is known as eNOS uncoupling. eNOS uncoupling can occur under several conditions, including cofactor tetrahydrobiopterin (BH4) deficiency, a shortage of the eNOS substrate l-arginine, the accumulation of the NOS inhibitor asymmetric dimethylarginine (ADMA), and increased eNOS S-glutathionylation [108]. BH4 is highly susceptible to oxidative reactions and can be oxidized to BH2 in a highly oxidative environment. Additionally, the peroxynitrite formed by the reaction between O_2_ and NO can oxidize BH4. As a result, BH4 depletion destabilizes NOS and induces its uncoupling, which, in turn, affects eNOS activity. Under conditions of reduced l-arginine availability, eNOS becomes less effective at converting it into NO, and this shift in activity can lead to eNOS uncoupling. ADMA is an endogenous inhibitor of eNOS. When ADMA levels are elevated, it competes with l-arginine to bind to eNOS, thereby reducing the availability of l-arginine for NO production.

#### 2.3.3. Cytochrome P450

CYP enzymes, a diverse group of heme monooxygenases, have been the focus of extensive research because of their central roles in the metabolism of drugs and xenobiotic compounds. Furthermore, they play crucial roles in the synthesis of sterols, fatty acids, eicosanoids, vitamins, and various other biochemical compounds, including ROS. Notably, upon modification by CYP enzymes, certain substrates give rise to reactive intermediates or products that can be implicated in the onset and progression of various diseases.

#### 2.3.4. Xanthine Oxidase

XOR plays a pivotal role in the final stages of purine catabolism, facilitating the oxidation of hypoxanthine to xanthine or subsequently xanthine to uric acid [109]. It exists in two interchangeable forms (depending on the types of substrate): (1) XO utilizes oxygen as a substrate, generating both O2^•−^ and H_2_O_2_, while (2) xanthine dehydrogenase is an NAD^+^-mediated type that utilizes NAD^+^ as a cofactor and does not generate ROS [110]. Importantly, oxygen concentration plays a critical role in determining whether H_2_O_2_ or O_2_^•−^ is generated [111]. In one specific study, XO treatment increased O_2_^•−^ and H_2_O_2_ levels in the XO inhibitor allopurinol treatment, preventing an increase in mitochondrial ROS levels in a model of cocaine-induced diastolic dysfunction [109].

### 2.4. ROS Signaling Pathways in ARDS

**Figure 2:**
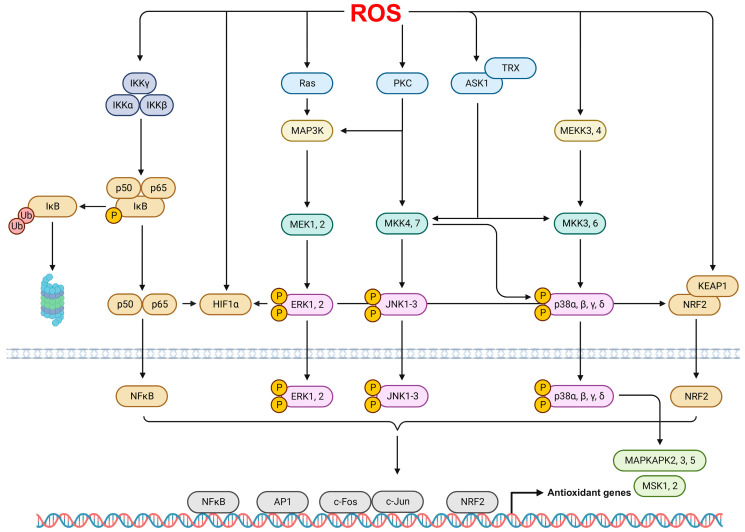
ROS-mediated signaling pathways. The signaling pathways involving ROS include the activation of NFκB and MAPK pathways. Specifically, ROS can activate NFκB through the phosphorylation of IκB and induce the phosphorylation of the MAPK pathway, which includes JNK, ERK, and p38 MAPK. Consequently, these activations promote the nuclear translocation of transcriptional factors such as NFκB, AP-1, c-Fos, c-Jun, and NRF2. These factors contribute to the regulation of genes associated with oxidant defenses.

In ARDS, ROS can be induced and activated through the activation of various signaling pathways, such as mitogen-activated protein kinases (MAPKs), protein kinase C (PKC), nuclear factor kappa B (NFκB), hypoxia-inducible factor 1 (HIF1), and activator protein-1 (AP-1), as shown in Figure 2.

MAPKs are serine-threonine protein kinases involved in various processes, such as cell growth, proliferation, and differentiation. They are categorized into subgroups, including extracellular signal-related kinases (ERKs), c-Jun N-terminal kinases (JNKs), and p38 MAPKs [112,113]. The ERK pathway regulates diverse cellular functions and is activated by various stimuli, such as growth hormones, oxidative stress, and inflammatory responses mediated by the phosphorylation of MAP/ERK Kinase (MEK) through MAPK kinase kinases (MAP3K) Raf [114]. Liu et al. [115] revealed that LPS stimulation augmented oxidative stress, inflammation, and neutrophil NETosis through the phosphorylation of Raf, MEK, and ERK in a murine lung inflammation model and neutrophils from patients with ARDS. Similarly, the elevated phosphorylation of ERK and JNK mediated excessive ROS production in models of hyperoxia-induced in vitro and in vivo ARDS [26].

JNK, also known as stress-activated protein kinase, is activated by physical or chemical stresses, cytokines, and ROS via the phosphorylation of upstream components such as MEK4, MEK7, and apoptosis signal-regulated kinase 1 (ASK1) [114,116]. ASK1 is inactivated upon binding to thioredoxin (TRX) in a resting state; however, when the thiol group of TRX is oxidized by H_2_O_2_ or other oxidants, it dissociates from ASK1, leading to the activation of JNK MAPKs [116,117]. Consistent with these observations, Sidramagowda et al. [118] showed that an increase in the phosphorylation of JNK and eukaryotic initiation factor 2α induced endoplasmic reticulum stress-mediated cell death in a murine model of hyperoxia-induced lung injury.

The p38 MAPKs encompass isoforms such as p38α, p38β, p38γ, and p38δ, and they are activated by a variety of stress responses, including inflammation, oxidative stress, heat, and physical shock [114,119]. Intermediaries of the p38 signaling pathway are phosphorylated by a range of stimuli, such as MEK3, MEK6, MEKK3, MEKK4, ASK1, transforming growth factor β-activated kinase 1, and dual-leucine-zipper-bearing kinase 1, allowing these molecules to crosstalk with various signaling pathways and responses [119]. A recent study showed that LPS-induced ROS and inflammatory cytokine production was significantly increased via the p38 MAPK and NFκB pathways in a murine ARDS model and RAW 264.7 macrophages [120]. Similarly, p38 MAPKs mediated increases in endothelial permeability following H_2_O_2_ exposure in bovine lung microvascular endothelial cells [121]. Additionally, Yuan et al. [122] demonstrated that MAP3K2 and MAP3K3 induced elevated ROS production through the phosphorylation of the NOX2 constituent p47^phox^ in ARDS models.

PKC comprises at least 12 isoforms, including PKCα, PKCβI, and PKCδ, and it possesses structural features sensitive to oxidative modification [123,124]. It regulates various cellular responses, such as movement, proliferation, and apoptosis. Notably, PKC contains a zinc-binding, cysteine-rich motif at the N-terminus regulatory domain that responds sensitively to oxidants such as H_2_O_2_, thereby becoming activated [113]. Bourdonnay et al. [125] showed that pathways such as PKCδ, p21-activated protein kinase-class I, and PI3K/Akt1 were associated with p40^phox^-mediated ROS production in rat alveolar macrophages (AMs) stimulated with IgG-opsonized *Klebsiella pneumonia*. Similarly, the inhibition of PKC attenuated hypochlorous acid-induced pulmonary artery pressure and vascular permeability in a rabbit lung injury model [126]. Additionally, increased sepsis-attributable mortality in mice deficient in nuclear factor erythroid 2-related factor 2 (NRF2) and gp91^phox^ was shown to be associated with LPS-induced ROS production (mediated by PKC activation) in murine AMs [127].

NFκB is a redox-sensitive transcription factor that regulates the expression of various genes associated with inflammation and immune responses [128]. It is composed of homodimers or heterodimers of five different subunits, including RelA/p65, RelB, cRel, p105/p50, and p100/p52, which contain the Rel homology domain essential for DNA binding, dimerization, and nuclear translocation [129,130]. Additionally, the Rel components also include a C-terminal transcription activation domain [130]. In the resting state, NFκB is retained in the cytoplasm as an inactive non-DNA binding form, regulated by IκB proteins that contain C-terminal ankyrin repeats, which inhibit DNA binding [128]. The activity of typical IκB proteins such as IκBα, IκBβ, and IκBε is regulated through phosphorylation via upstream IκB kinases (IKK) and subsequent ubiquitination by E3 ubiquitin ligases [113,128]. After partial processing by the 26S proteasome, the NFκB dimers are translocated from the cytoplasm to the nucleus, where they bind to promoters and activate target genes [113,129]. Exogenous ROS stimuli such as H_2_O_2_ typically regulate the activation of NFκB signaling pathways through the phosphorylation of tyrosine residues, particularly Tyr42, on IκBα [131]. Ghobadi et al. [132] showed that NFκB potentially mediates elevated serum ROS production while decreasing antioxidant capacity in patients with COPD. Concordantly, a recent study showed that LPS-induced NO and inducible NO synthase (iNOS) were regulated by the phosphorylation of NFκB p65 in a murine respiratory inflammation model [133]. Similarly, particulate matter-elicited oxidative stress, intracellular ROS levels, and related inflammatory responses and apoptosis were increased via NFκB and MAPK signaling pathways in [134]. Additionally, Lin et al. [135] revealed that PPARγ, which has antioxidative and anti-inflammatory functions, attenuated the NFκB-mediated recruitment of neutrophils to the lung, ROS production, and inflammatory gene expression in a murine lung inflammation model after LPS stimulation.

HIF1 is a conserved family member of the heterodimeric basic helix-loop-helix transcription factors; it is composed of oxygen-sensitive α subunits, including HIF1α, HIF2α, and HIF3α, and three constitutively expressed β subunits, including HIF1β, HIF2β, and HIF3β [136]. HIF1 accumulates in response to low oxygen levels, various cytokines, and inflammatory mediators [137]. HIF1α is a major regulator of macrophage M1 polarization and the expression of glycolytic genes such as glucose transporter 1, hexokinases, fructose-2,6-biphosphatase 3, and lactate dehydrogenase A [138,139]. Succinate, which is a Krebs cycle metabolite whose production is increased in response to enhanced glycolysis, inhibits the prolyl hydroxylases (PHDs) involved in the polyubiquitination and proteasomal degradation of HIF1α, thereby leading to the stabilization and accumulation of HIF1α and the production of inflammatory cytokines such as IL1β [139,140,141]. Additionally, increased ROS production and accumulation lead to the stabilization of HIF1α through reduced PHD activity in JunD-deficient cells [142]. A recent study showed that NFκB and HIF1α enhanced ovalbumin (OVA)-induced ROS generation, airway resistance, and mucus production in an allergic airway disease model [143]. Similarly, Sun et al. [144] showed a decrease in the activity of antioxidative enzymes including catalase, glutathione peroxidase, and SOD mediated by HIF1α and NFκB in models of LPS-induced murine lung injury.

AP-1 is a dimer comprising members of the JUN (c-Jun, JunB, and JunD), FOS (c-Fos, FosB, and Fra-1/2), ATF (ATF2/3/4/5/6B/7, B-ATF, B-ATF2/3, and JDP1/2), and MAF (MafA, MafB, c-Maf, Nrl, and MafF/G/K) families, which contain a basic leucine zipper (bZIP) domain necessary for DNA interaction and the formation of homodimers and heterodimers [145]. Further, AP-1 dimers collaborate with transcription factors such as transcription coactivators CREB binding protein/p300 and NFκB p65, which are absent from a bZIP domain, to contribute to gene expression [146]. AP-1 is activated in response to cytokines, hormones, growth factors, and ROS and plays an essential role in immunoglobulin production, cell proliferation, differentiation, and apoptosis [146]. Notably, ROS induce the expression of AP-1 and AP-1-dependent genes, contributing to relevant signal transduction related to redox cycling [147,148]. Additionally, AP-1 and ROS are involved in the pathogenesis of lung inflammation. Kim et al. [149] showed that the pharmacological inhibition of AP-1 and NFκB, through ROS scavenging, attenuated OVA-induced lung inflammation and airway hyperresponsiveness in a murine allergic asthma model. Similarly, activated AP-1, via the phosphorylation of c-Jun, has been shown to interact with ROS, leading to an increase in leukocyte infiltration and cyclooxygenase 2 (COX2) expression in a leptin-induced lung inflammation model [150].

## 3. Therapeutic Approaches Targeting ROS in ARDS

In ARDS, the permeability of the alveolar-capillary barrier increases, causing the edema fluid containing a large amount of protein to flow into the lung parenchyma, leading to epithelial cell dysfunction and reduced lung compliance and gas exchange [151,152]. Recently, in a murine lung injury model, Jiang et al. [153] showed that NOX4 activation and ROS production inhibit the expression of endothelial cell tight junction proteins zonula occludens-1 and occludin, leading to endothelial cell barrier dysfunction. Such damage and dysfunction with respect to the endothelium and epithelial cells lead to pulmonary edema and augmented inflammatory factors and oxidative stress, thereby amplifying the inflammatory response by inducing the activation and chemotaxis of leukocytes and lymphocytes [154]. Pulmonary endothelial and epithelial cells, particularly macrophages and neutrophils that exhibit phagocytosis, are the major factors in the production of large amounts of ROS mediated by NOX [155,156]. In addition, ROS, MPO, neutrophil elastase, and azurophilic granules released via NETosis, a process of extracellular release from activated neutrophils, also contribute to proteolytic and oxidative tissue damage and inflammatory responses [157,158]. From this perspective, strategies to suppress the excessive production of ROS and related signaling mechanisms in cells and tissues which are mainly involved in the pathology of ARDS will have a positive effect on the treatment of ARDS.

### 3.1. Role of the NRF2 Pathway in ARDS

NRF2 is a transcriptional regulator of genes with antioxidant and anti-inflammatory effects that has emerged as a major target in the treatment of ARDS. As a master regulator, NRF2 crosstalks with multiple signaling pathways, controlling the activity of various antioxidant enzymes and oxidizing enzymes such as NOX, NO synthase, XOR, and CYP [159,160]. Upon sensing oxidative stress, NRF2 translocates from the cytoplasm to the nucleus; it is activated by upstream signaling molecules such as Akt, AMP-activated protein kinase (AMPK), NFκB, and glycogen synthase kinase 3β (GSK3β) [160].

The phosphorylation of Akt induces the nuclear translocation of NRF2, leading to the up-regulation of heme oxygenase-1 (HO-1) and NAD(P)H quinone oxidoreductase 1 through the PI3K/Akt pathway. This process reduces oxidative stress and inflammation mediated by forkhead box protein O1-NLRP3, reducing NLRP3 inflammasome activity in the lungs [161]. The phosphorylation of AMPK increases the production of NRF2-induced antioxidant enzymes, thereby alleviating inflammatory lung injury by suppressing inflammatory responses and oxidative stress mediated by the NLRP3 inflammasome [162]. Additionally, NRF2 activation results in the suppression of inflammation and oxidative damage; this is mediated by the inhibition of IKK/IκB phosphorylation and the nuclear translocation of the p65 subunit of the redox-sensitive transcription factor NFκB [162]. Upon enhancing NRF2 activation and repressing NFκB pathways, inflammatory responses were suppressed in ischemic reperfusion-induced lung injury [163] and auranofin-stimulated AMs [164].

Kelch-like ECH-associated protein 1 (Keap1) features a broad-complex, tramtrack and bric-a-brac domain (BTB) that engages with the Cul3-Rbx1-E3 ligase complex, governing Keap1 homodimerization. Additionally, it possesses a double glycine repeat domain (DGR) that binds to the ETGE and DLG motifs within the NRF2-erythroid-derived CNC homology domain (Neh) 2 of NRF2, orchestrating the ubiquitination and degradation of NRF2 [165,166,167]. Keap1 primarily modulates the stabilization and transcriptional activity of NRF2 through its BTB and DGR domains. Normally, NRF2 undergoes swift ubiquitination by Keap1, leading to its subsequent degradation by the proteasome. However, when exposed to various stresses such as ROS, interactions with Keap1 are disrupted, reducing proteasomal degradation and enhancing nuclear translocation [166]. GSK3β is involved in an altered Keap1-independent NRF2 degradation mechanism. It phosphorylates motifs within the Neh6 domain of NRF2, initiating proteasomal degradation mediated by the dimeric β-transducin repeat-containing protein [166,168]. Only a few NRF2 molecules that are not ubiquitinated and degraded translocate to the nucleus, activating the expression of antioxidant-related factors such as HO-1, glutamate-cysteine ligase modifier subunit, glutamate-cysteine ligase catalytic subunit, glutathione peroxidase (GPx), and SOD3 [169,170].

Recent studies have shown that single-nucleotide polymorphisms (SNPs) in NRF2 correlate with the severity of ARDS. In one specific study, the hierarchical clustering of 72 inbred strains of mice identified a significant association between NRF2 haplotypes and hyperoxic lung injury phenotypes [171]. Additionally, the −617(C/A) SNP (rs6721961) within the proximal promoter of NRF2 is correlated with an increased risk of ARDS [172]. Similarly, NRF2 rs6721961 exhibited a nominal correlation with the severity of ARDS outcomes in Caucasian subjects with systemic inflammatory response syndrome [173]. Consistent with these findings, the augmented nuclear translocation of NRF2, mediated by the phosphorylation of AKT [161], AMPK [174], NFκB [175,176], and GSK3β [162], has been shown to result in the suppression of ROS production and various inflammatory responses, including inflammatory lung damage, elevated inflammatory gene expression, and the activation of the NLRP3 inflammasome, in murine lung injury models. Additionally, Cen et al. [177] demonstrated that NRF2 ameliorates mitochondrial dysfunction, endothelial disruption, the apoptosis of endothelial cells, and lung inflammation in mice. More recently, Wei et al. [178] revealed that NRF2 provides protection against LPS-induced ARDS in mice. NRF2 mitigates LPS-induced histopathological symptoms, suppresses inflammatory gene expression, and notably promotes macrophage M2 polarization through the activation of PPARγ while inhibiting the phosphorylation of NFκB p65 [178]. In summary, these observations reinforce the status of NRF2 as a potentially effective target for the prevention and treatment of ARDS, as it suppresses inflammatory responses, mitigates oxidative stress, and regulates diverse immune responses associated with various immune cells, such as macrophages and endothelial cells.

### 3.2. Antioxidant Therapies

Maintaining redox homeostasis and curbing excessive ROS production throughout the development, progression, and exacerbation of ARDS presents a promising avenue for alleviating and treating clinical symptoms. This can be achieved by regulating multiple signaling pathways implicated in ARDS pathogenesis. From this standpoint, antioxidant enzymes like SOD, glutathione (GSH), and various pharmacological and nonenzymatic antioxidants have been explored for their therapeutic potential in ARDS.

SOD catalyzes the conversion of O_2_^•−^ into H_2_O_2_ and is categorized into cytosolic copper-zinc SOD (CuZnSOD), mitochondrial manganese SOD (MnSOD), and extracellular SOD (ECSOD), found in fibroblasts and endothelial cells [160]. While investigating therapeutic approaches to ARDS, researchers have focused on SOD’s ability to reduce and terminate intracellular ROS production [179,180,181]. Notably, in one study, the intratracheal delivery of recombinant human CuZnSOD yielded a reduction in the levels of acute inflammation markers in tracheal aspirate fluid, coupled with increased antioxidant activity and concentration in preterm neonates with respiratory distress syndrome [182]. Research has also explored the use of conjugated antioxidant enzymes with polyethylene glycol (PEG) or lecithin, revealing enhanced delivery to cells, protection against oxidative stress, and a reduction in the severity of ARDS [183,184]. A synthetic Mn-containing SOD mimetic, as shown by Ndengele et al. [185], suppressed LPS-induced O_2_^•−^ and inflammatory cytokine production in murine AMs. Additionally, commercialized SOD mimetics, such as manganese(III)tetrakis(1-methyl-4-pyridyl)porphyrin, demonstrated the alleviation of oxidative and nitrative stress-mediated MPO activity and vascular permeability in a murine lung inflammation model following LPS stimulation [186]. Similarly, the synthetic salen-manganese complex EUK-8, exhibiting SOD and catalase activities, attenuated clinical symptoms of ARDS induced by LPS in a porcine model, as revealed by Gonzalez et al. [187]. Another SOD and catalase mimetic, EUK-134, encapsulated by PEG, exhibited enhanced protection against pulmonary edema in a murine lung inflammation model compared to its counterpart without EUK following LPS treatment [188]. Furthermore, a synthetic SOD mimetic, MnTBAP, repressed inflammatory gene expression, O_2_^•−^ production, lung tissue damage, and necrosis in a murine lung inflammation model, as demonstrated by Suresh et al. [189].

GSH plays a pivotal role in scavenging intracellular H_2_O_2_, with GPx being the major enzyme responsible for this reducing mechanism in the redox cycle [190,191]. GPx catalyzes the oxidation of glutathione using H_2_O_2_ or lipid hydroperoxides (LOOH), with reduced GSH serving as an electron donor [192]. Glutathione disulfide (GSSG), formed during the reaction, is subsequently reduced back to GSH by glutathione reductase, utilizing the NADPH generated through the pentose phosphate pathway as an electron donor [160,193]. GPx, predominantly distributed in the cytoplasm and mitochondria, contains a selenium atom at its active site (usually in the form of selenocysteine) [194]. Additionally, free GSH functions as a water-soluble antioxidant by directly interacting with radical intermediates in non-enzymatic catalytic reactions [194]. The GSH-mediated O_2_^•−^ scavenging induces a radical propagation reaction involving several steps, leading to the formation of thiyl radicals (GS·) and H_2_O_2_ [194,195]. In a murine lung injury model, GSH attenuated LPS-induced cellular apoptosis, mitochondrial dysfunction, and oxidative stress while restoring reduced levels of SOD2 protein [196]. Kim et al. [197] demonstrated that enhanced GPx activity, mediated by selenium treatment, reduced lung injuries and lipid peroxidation in paraquat-intoxicated rats. Furthermore, the administration of an organoselenium compound, Ebselen, enhanced antioxidant activity and suppressed inflammatory responses, including MPO activity, leukocyte infiltration, and lipid peroxidation, in a murine model of carrageenan-induced lung inflammation [198]. In a study by Britt et al. [199], pharmacological TRX reductase-1 aurothioglucose alleviated lung injury and mortality while increasing GSH activity in a murine ARDS model following LPS and hyperoxia exposure. Interestingly, Moute et al. [200] demonstrated that a synthetic GPx-containing selenium mimetic, BXT-51072, and BXT-51077 suppressed neutrophil-mediated inflammatory responses and endothelial alterations.

Synthetic and pharmacological antioxidants play a crucial role in preventing ARDS development by inhibiting intracellular ROS production and associated oxidases. Notable antioxidants, such as apocynin, GKT137831, tempol, sivelestat, and NAC, have been developed for this purpose. Apocynin, a NOX inhibitor, demonstrated the attenuation of acute hypoxia-induced gp91^phox^ expression and O_2_^•−^ production in porcine lung arteries, thereby mitigating the progression of ARDS [201]. Muzaffar et al. [202] showed that apocynin and iloprost inhibited XOR-induced gp91^phox^ expression and O_2_^•−^ formation in porcine pulmonary artery endothelial cells. The NOX1/4 dual inhibitor GKT137831 ameliorated lung injuries and suppressed the expression of inflammation- and autophagy-related genes in a murine ischemic lung injury model [203]. In recent studies, treatment with the commercialized free radical and NO scavenger TEMPOL resulted in reduced intracellular ROS production and increased SOD and catalase activity in the human pulmonary epithelial cell line Calu-6 [204]. Sivelestat sodium, a NOX inhibitor, suppressed pathological symptoms of ARDS, intracellular ROS, and MDA production while increasing SOD and GPx levels in A model of *K. pneumonia*-induced murine lung injury [205]. Additionally, sivelestat sodium promoted HO-1 production via the translocation of NRF2 to the nucleus in human pulmonary microvascular endothelial cells [205].

NAC, in its acetylated form of cysteine, possesses antioxidant capabilities by virtue of its sulfhydryl functional group (-SH) [206]. It acts as a critical cysteine supplier in the synthesis of glutathione, a precursor that elevates glutathione concentration within the cell [206,207]. NAC also forms complexes that facilitate the excretion of various toxic heavy metals and transition metals, such as Cd^2+^, Hg^2+^, and Pb^2+^, as well as Cu^2+^ and Fe^3+^, through the free thiols that bind with redox metal ions [208]. Clinical studies have highlighted the efficacy of NAC in preventing and treating inflammatory lung diseases such as COPD, ALI, and ARDS. Tse et al. [209] demonstrated that administered NAC improved small airway functions and alleviated exacerbation frequency in patients with COPD. In experimental settings, NAC treatment reduced lung injury, neutrophil infiltration, oxidized GSH levels, and breath H_2_O_2_ levels in rats following intratracheal IL1α administration [210]. Kolomaznik et al. [211] observed that administered NAC markedly improved ventilatory parameters while suppressing leukocyte migration, edema, oxidative stress, and inflammation in a murine lung injury model requiring mechanical ventilation following LPS stimulation. Additionally, Ortolani et al. [212] showed that NAC supplementation holds therapeutic potential for ARDS by enhancing GSH levels while reducing MDA and ethane concentration in the epithelial lining fluid of patients with ARDS.

Nonenzymatic antioxidants such as vitamins and flavonoids have been explored as treatments for ARDS by inhibiting intracellular ROS production and associated oxidases. Vitamins, serving as coenzymes, metabolic activators, cellular components, and ROS scavengers, play essential roles in combating oxidative stress [213]. Notably, vitamins C (ascorbic acid) and E (α-tocopherol) have garnered attention for their radical scavenging properties, activation of antioxidant enzymes, and regeneration of antioxidants under oxidative stress conditions [213,214]. Vitamin C, a water-soluble antioxidant, exists in forms such as l-ascorbic acid and l-dehydroascorbic acid [215]. It effectively eliminates active oxygen species, including reactive NO and H_2_O_2_. Studies have demonstrated the beneficial effects of vitamin C supplementation in various ARDS models [215]. Dioxygenase utilizes ascorbic acid as a co-substrate for substrate reduction [216]. Fisher et al. [217] showed that intraperitoneally delivered vitamin C ameliorated LPS-induced pulmonary inflammation and procoagulant activity in a murine septic lung injury model. Similarly, vitamin C supplementation attenuated sepsis-associated coagulopathy and inflammatory responses, enhanced epithelial barrier function, and improved alveolar fluid clearance in a murine abdominal sepsis-associated ARDS model [218]. Kearns et al. [219] revealed that vitamin C supplementation suppressed ischemic-reperfusion-induced MPO activity, lung neutrophil recruitment, and microvascular leakage in a murine ARDS model. Additionally, Patel et al. [220] demonstrated that vitamin C reduced oxidative and nitrosative stress, leukocyte infiltration, and HMGB1 protein-mediated macrophage dysfunction while improving lung integrity in the murine hyperoxia-induced ARDS model.

The most abundant and potent form of vitamin E, a liposoluble antioxidant consisting of eight isoforms, is α-tocopherol [221]. α-tocopherol’s chroman head group exhibits major antioxidant activity, preventing chain oxidation reactions and suppressing damage to lipid membranes [160]. The chroman head group of α-tocopherol demonstrates significant antioxidant activity by donating its hydrogen to the peroxyl radical, forming an α-tocopheroxyl radical. This process prevents chain oxidation reactions, thereby suppressing the oxidation of cell membranes and protecting lipid membranes from damage [222]. Vitamin E can be regenerated and reused by other antioxidants such as vitamin C [223]. Studies have explored the benefits of vitamin E isoforms in various contexts. One specific study showed that supplementation with the vitamin E isoform γ-tocopherol decreased LPS-elicited sputum eosinophils and mucins and neutrophil recruitment in the airways in a double-blind, placebo-controlled study for volunteers with mild asthma [224]. Similarly, γ-tocopherol has been shown to alleviate the LPS-induced infiltration of inflammatory cells, mucin production, and inflammatory gene expression in a murine lung inflammation model [225]. Rocksén et al. [226] demonstrated that administering vitamin E suppressed lung edema, lung injuries, neutrophil recruitment to airspaces and the interstitium, and transendothelial migration in a model of LPS-induced murine lung injury. Additionally, vitamin E reduced plasma nitrotyrosine and conjugated diene levels, increased airway pressure and pulmonary permeability index, and improved the PaO_2_/FiO_2_ ratio in a lung inflammation model [227].

α-Lipoic acid (α-LA), a naturally synthesized short-chain fatty acid with sulfhydryl groups, and its reduced form, i.e., dihydrolipoic acid, are known as universal antioxidants that function in both hydrophobic membranes and hydrophilic cytosol [228]. α-LA is involved in metal chelation, free radical quenching, and the recycling of other antioxidants such as vitamin C, E, and glutathione (GSH) [229]. Studies have highlighted the antioxidant properties of α-LA in various models. Zhang et al. [230] showed that α-LA repressed LPS-induced NFκB activation and related inflammatory gene expression in mice hearts, aortae, and lungs. Additionally, intraperitoneally delivered α-LA significantly enhanced GSH production and catalase and GPx activities while repressing serum MDA production in a model of oleic acid-induced murine lung injury [231]. α-LA treatment decreased pathological ARDS symptoms such as mononuclear cell infiltration, vascular permeability, and edema in lung tissues [231]. Recently, Shoji et al. [232] demonstrated that an α-LA derivative, the dihydrolipoyl histidinate zinc complex, attenuated the pathology scores of lung injury and suppressed inflammatory cell infiltration, gene expression, and NFκB p65 concentration in the BALF of a murine ARDS model following LPS stimulation.

Flavonoids, a class of polyphenols, exhibit anti-inflammatory, antioxidant, metal ion chelation, and free radical scavenging activities, countering oxidative stress by mitigating lipid oxidation and intracellular ROS [233]. Comprising aglycone and glycoside forms, flavonoids demonstrate higher antioxidant and antimicrobial activities in the glycoside form [234]. Structurally, flavonoids consist of two phenyl rings and a heterocycle, with various attached functional groups [233]. They are grouped into subgroups such as flavones, flavonols, flavanones, flavanols, isoflavonoids, anthocyanins, and chalcones based on their heterocyclic structures, each exhibiting diverse biological activities [233]. In a study by Kuo et al. [235], luteolin pretreatment improved the histopathological symptoms of ARDS and reduced oxidative damage, lipid peroxidation, and inflammatory gene expression in a murine model of LPS-induced ARDS. Administered luteolin also enhanced alveolar fluid clearance and reduced lung edema through the promotion of cyclic guanosine monophosphate/PI3K-mediated epithelial sodium channel expression in an ARDS murine model after LPS stimulation in [236]. Takashima et al. [237] demonstrated that intratracheally delivered quercetin alleviated LPS-induced wet lung-to-body weight ratios, metalloproteinase 9 activity, and the expression of inflammatory genes such as IL1β, IL6, and TNFα via a HO-1 dependent pathway in a murine lung injury model. Similarly, quercetin has been shown to decrease endotoxin-induced NO, MPO activity, MDA levels, lung permeability, leukocyte infiltration, and the expression of inflammatory genes such as IL1β, IL6, TNFα, COX2, HMGB1, and iNOS in a murine septic lung injury model [238]. Xanthohumol attenuated oxidative stress and inflammatory gene expression in LPS-induced ARDS by upregulating the NRF2 pathway through the activation of AMPK/GSK3β while downregulating the NFκB pathways [162]. Additionally, Shen et al. [239] demonstrated that epigallocatechin-3-gallate reduced hemorrhages, alveolar edema, inflammatory cell recruitment, and the expression of inflammatory genes such as TNFα, IL1β, and IL6 via Toll-like receptor-mediated NFκB activation in a model of paraquat-induced murine lung inflammation.

## 4. Conclusions

ARDS results in sudden and severe lung failure; despite elevated mortality rates, the syndrome is currently managed primarily through supportive care due to the absence of specific targeted treatments. The identification of increased levels of ROS in ARDS models and the clinical correlation between mortality and oxidative stress highlights the role of ROS as a potential target for ARDS treatment. Inhibiting ROS signaling through antioxidants has emerged as a prospective therapeutic approach for patients with ARDS. Nevertheless, conducting additional research, particularly through clinical trials, is imperative for comprehensively assessing the therapeutic efficacy of antioxidants.

## Figures and Tables

**Table 1:** ROS production in preclinical models of ARDS.

Candidate	Inducer	Conc.	Time	Animal	Organ	Method	Pathway	Ref
Neferine	LPS (i.p.)	20 mg/kg	6 h	C57BL/6	Lung	DCFH-DA	NFκB	[80]
Abscisic acid	LPS (i.t.)	4 mg/kg	2 day	C57BL/6	Lung	DCFH-DA	PPARγ	[81]
Berberine	LPS (i.p.)	20 mg/kg	6 h	C57BL/6	Lung	DCFH-DA	NFκB	[82]
Carnosine	LPS (i.t.)	1 mg/kg	24 h	ICR	Lung	In vivo imaging		[83]
Fraxin	LPS (i.p.)	20 mg/kg	6 h	C57BL/6	Lung	DCFH-DA	NFκB, MAPK	[84]
MH	LPS (i.n.)	5 mg/kg	25 h	C57BL/6	BALF	DCFH-DA	p38 MAPK, NFκB	[85]
EA	LPS (i.p.)	5 mg/kg	3 day	C57BL/6	Lung	DHE		[31]
Fasudil, NipR1, T-SSP	LPS (i.t.)	2 mg/kg	6 h	C57BL/6	Lung	EPR	NLRP3	[86]
Benziodarone	LPS (i.t.)	1 mg/kg	24 h	ICR	Lung	IHC(8-OHdG)	α7nAchR	[87]
eNAMPT-neutralizing antibody	LPS/VILI (i.v.)	25 μg/kg/100% O_2_ (12 h)		Minipig	Lung	EPR	NFκB, Akt/mTORC2	[88]
Aspirin	Hyperoxia	99% O_2_(72 h)		FVB/NJ	BALF	DCFH-DA	NFκB	[89]

i.p., intraperitoneal; i.t., intratracheal; i.n., intranasal; i.v., intravenous; DCFH-DA, 2′,7′-dichlorofluorescein diacetate; DHE, dihydroethidium; EPR, electron spin resonance; IHC, immunohistochemistry; 8-OHdG, 8-hydroxy-2′-deoxyguanosine; PPARγ, peroxisome proliferator-activated receptor γ; α7nAchR, α7 nicotinic acetylcholine receptor; MH, methyl p-hydroxycinnamate; EA, electroacupuncture; NipR1, nitration inhibitory peptide for RhoA; T-SSP, antioxidant Tiron; eNAMPT, extracellular nicotinamide phosphoribosyl transferase.

## Data Availability

Not applicable.
